# Antiseptic Surgery

**Published:** 1879-06

**Authors:** G. B. Pratt

**Affiliations:** Elkhart, Ind., Late Resident Physician of the King’s County Hospital, Brooklyn


### ARTICLE V.

ANTISEPTIC SURGERY. By G. B. PRATT, M.D., Elkhart, Ind., late Resident Physician of the King's County Hospital, Brooklyn.

It would be very interesting, and demonstrate in a very forcible manner the world-wide interest taken in the subject of this article, could a collection be made of all the papers, pamphlets and books bearing on this subject, which have been written and published during the past few years. What a mass of literature, speaking in every language both *pro* and *con*, showing the spirit of advancement and the desire for a more perfect system, for better results, which animate the surgeon of to-day! And it may be asked, what subject is of more importance or of greater interest to the surgeon and the general practitioner as well, than that of the management of wounds? Are not the well-being and life of his patients, as well as his own reputation, often dependent upon the results of such treatment? It is not very remarkable, therefore, that any method which claims to diminish the mortality and expedite the cure of these cases, should receive a ready welcome from every member of the medical profession.

Every innovation in surgery, though possessed, perhaps, of some good qualities, is attended by a host of useless and oftentimes injurious properties, but practical experience, attended by the sharp criticisms of practical minds, tends ere long to separate the wheat from the chaff, and while this is left to the winds, that is gathered into our store-houses of knowledge and carefully treasured for the use of future generations, until in the light of a more advanced knowledge that which we pronounced good will be again refined.

But there are certain great principles in surgery which have ever been, and always will be, the sure guides to the surgeon in the management of wounds, and any measure which will aid us in carrying out the more perfectly these rules, will command our respect and commendation. The cardinal principles in the treatment of wounds to which I refer are these: coaptation, drainage, cleanliness and rest, and these in order to be effective must be perfect, each in its way. There must be perfect coaptation, perfect drainage, perfect cleanliness and perfect rest; if one of these indications be neglected, the others will be unable to supply the deficiency, and thus their efforts at repair will prove abortive. As to what extent the "antiseptic method" will aid us in fulfilling these indications, or how essential the different steps of the method really are, are questions which the ablest minds in our profession are discussing at the present time.

To Mr. Joseph Lister undoubtedly belongs the honor of having carefully developed this method and conscientiously followed its teachings to the most minute proceeding, but he deserves the condemnation passed upon him by the Clinical Society of London for not making his results known, he having published but a few cases, and these mainly to illustrate his method. A writer in the *New York Medical Journal* says, "it seems to me the name of Joseph Lister must outrank in medicine all of his century, not excepting the discoverer of anæsthesia."

There are several antiseptic methods, all of which differ in some respects from that of Lister. As all are aware, Lister insists upon the use of the carbolic acid spray continuously. Mr. Callender uses no spray, but having tied the bleeding vessels with carbolized catgut, he washes out the stump with a carbolic solution of 1 to 20, and secures drainage by two pieces of carbolized gutta-percha, closing the wound with silver sutures. Over this he places a few layers of carbolized lint, covered by a sheet of gutta-percha, next a thick layer of cotton-wool, and finally a bandage. Rest is secured by a stump splint, hinged to allow ready re-dressing. His results after capital operation have never been equaled.

Mr. Holmes has followed Lister in all the details excepting the spray only, and the results obtained in his practice, including treatment of compound fractures, have been quite remarkable. Mr. Gamgee prefers treating wounds by dry and infrequent dressings, rest and pressure. The edges of the wound are brought in perfect coaptation by silver sutures, a gauze and oakum pad placed over the wound and drainage secured by means of rubber tubing; cotton wool is then placed over all, and a splint applied in treating a limb and perfect rest secured. The dressings are not removed, unless demanded, until the ninth day. He reports excellent results. In these methods cited, primary union is aimed at, the same as by the Lister plan.

Dr. James R. Wood has made known the success which may attend the use of a method first introduced in the early part of this century by Kern, a Vienna surgeon. In this, which is called the open treatment, and the cotton wool dressing of Guérin, there is no attempt made at primary adhesion. Dr. Wood leaves the wound entirely open; perfect drainage is thus secured, and the parts are kept scrupulously clean by the frequent and free use of carbolic acid solution. He has published through his house-surgeon fourteen cases of amputation treated in this way without a single death.

M. Jules Guérin insists upon perfect occlusion with compression. He thoroughly washes the wound with carbolized water, packs in small masses of raw cotton, and, surrounding the stump with two or three layers of cotton wool, applies a linen band tightly over all, then another layer of cotton, then another band, and so on until he has used several pounds of cotton wool. In a case in which he applied it, at St. Bartholomew's Hospital, London, he used up five pounds of cotton wool. He claims very flattering results from his dressing.

In Dr. Wood's method we follow two of the rules of surgery of which I have spoken, viz.: cleanliness and drainage; and rest may also be secured to a certain extent. Cases treated in this manner require a comparatively long time for healing, and are apt to become very tedious. This method illustrates what may be accomplished by meeting two of the indications perfectly, and would go to show that this spray was not absolutely indispensable to very good results under very bad circumstances. And when we remember that the ward in which his patients were treated had been vacated the previous year in consequence of puerperal fever, we will the better realize the disadvantages he had to contend with. Fourteen consecutive amputations and *no deaths*.

The question naturally arises in the mind of every surgeon, after reading such a report: Is the spray an essential factor to the most perfect result in the treatment of wounds? In other words, is the spray any benefit whatever, or is it simply a superfluous part of the Lister method, which may as well be dispensed with. If it is of no value, why burden ourselves with its use? In an editorial on this subject in a recent number of the *Medical Record* the writer says,* "There is a growing conviction that the spray can be dispensed with, and that a thorough washing of the cut surfaces with an antiseptic fluid will accomplish the same end. If we were called upon to decide what was the most important element in Mr. Lister's dressing, we should say that it was his system of drainage, and to this, more than anything else, must be attributed his success." Billroth says,† "Scrupulous cleanliness and very careful draining away of all the discharges from the wound are by far the most important factors in the success of this method."

Bearing on the same subject Dr. Gross writes,‡ "The treat-

*The *Medical Record*, March 1st, 1879, page 208,

†Billroth's Surgery, vol. 1, page 142.

‡Gross' System of Surgery, vol. 1, page 371.

ment of open wounds, of whatever character, is often greatly expedited by the employment of protective measures, not too frequently changed. The much-vaunted carbolic acid treatment of Mr. Lister owes, I have no doubt, more of its efficacy to this circumstance than to any special virtue of the acid itself." But statistics, in a discussion of this character should, and do, have more weight than the unsupported opinions of the best surgeons. In the mortality table prepared by Dr. R. F. Weir,* the per cent. of deaths following amputation of the thigh, leg, arm and forearm was 2.27 under Callender's treatment (without the use of the spray), and after the method of Lister, reported by Volkmann, the per cent. was 3.09. The results attending Callender's management of wounds has never been equalled.

As an additional argument that the spray may be done away with, I would refer to the success of Mr. Lister himself, in his treatment of compound fractures; by simply washing out the wound with a carbolized solution he obtains as good results as by the use of the spray in operations. Besides the expense attending Lister's plan, those who have tried it will readily admit the great difficulty met with in trying to follow his directions exactly. There is sure to be some mishap during the operation, some instrument or some assistant's hands failed of being washed with the antiseptic solution, or the spray would be for an instant misdirected. Now as the germ theory constitutes the basis of Lister's antiseptic method, success in its practice can only be looked for with the aid of attention to minutiæ and details, and to extreme care in manipulation. One single cystic germ making its way to the surface of the wound would, in accordance with this theory, be sufficient to light up all the mischief which it is the object of this method to avert. Is it any wonder, then, that surgeons become discouraged, and question the necessity of a proceeding which is the occasion of so much annoyance, and which, though conducted with the utmost care, leaves very little hope that the effort has been successful?

I would not limit myself to a one-sided presentation of this question, being well aware there are many of the leading minds

---

* *New York Medical Journal*, January, 1878, page 51.

in our profession who strongly advocate Lister's system, in all its details, and who attribute their success to a faithful observance of the rules as laid down by him. In a letter from Berlin, in a recent number of the MEDICAL JOURNAL AND EXAMINER, the writer says of Langenbeck, " With the use of the antiseptic spray, he says that time has ceased to be an essential factor in the performance of most surgical operations."

Erichsen, after speaking of certain morbid conditions of the blood and constitutional derangements of the patient, says,* " It is undoubtedly these disturbing influences that prevent the complete success of Lister's method, in a certain number of cases. Theoretically, it is perfect, in practice its success is not constant."

A writer in the *London Lancet* remarks,† " The following cases may prove, I think, of interest to those already engaged in the practice of antiseptic surgery, or who may be (as I myself was for long) on the borderland of belief regarding the soundness of the doctrine on which it was based; a position from which I was ultimately driven by what I saw and heard during a long course of visits to Professor Lister's wards in the Edinburg Royal Infirmary."

I think, after all has been said and done, the homely adage (if I may be permitted to use it) that " every dog has his day," will be found to be just as true regarding Lister's antiseptic method as any new method for the treatment of disease, whether surgical or medical, which has ever been introduced, and that a modification of this plan will be fixed upon which will insure the closest observance of the four cardinal principles of the treatment of wounds. To this end the efforts of every surgeon should be directed. Let us strive to simplify rather than complicate surgical procedures, and it is a noteworthy fact that this is the tendency of the age; a healthy sign, and one which gives promise of grand results in the future.

ELKHART, Ind., April, 1879.

---

*Erichsen's Surgery, vol. 1, page 174.

†Illustrations of Antiseptic Treatment in Minor Surgery. By J. C. O. Will, M.D. Am. Reprint October, 1878, page 443.

